# Longitudinal Evaluation of Renal Function in Patients with Acquired Solitary Kidney—Urological Perspectives Post-Nephrectomy

**DOI:** 10.3390/jcm13237470

**Published:** 2024-12-08

**Authors:** Marius Ivănuță, Dragoș Puia, Diana Carmen Cimpoeșu, Ana-Maria Ivănuță, Ovidiu Daniel Bîcă, Cătălin Pricop

**Affiliations:** 1 “Grigore T Popa”, Faculty of Medicine, University of Medicine and Pharmacy, 700115 Iasi, Romania; marius_ivanuta@yahoo.com (M.I.);; 2 Department of Urology, “Dr. C.I. Parhon” Clinical Hospital, 700503 Iasi, Romania; 3 Center for Morphological and Spectroscopic Analysis of Urinary Stones “Michel Daudon”, 700503 Iasi, Romania; 4 Department of Emergency Medicine, “Grigore T. Popa” University of Medicine and Pharmacy, 700115 Iasi, Romania; 5 Emergency Department, “St. Spiridon” Emergency Clinical County Hospital, 700111 Iasi, Romania; anamariaion92@yahoo.com; 6 2nd Department of Surgery—Pediatric Surgery and Orthopedics, “Grigore T. Popa” University of Medicine and Pharmacy, 700115 Iasi, Romania; ovidiu-daniel.bica@umfiasi.ro

**Keywords:** chronic kidney disease, acquired solitary kidney, nephrectomy, lithiasis, renal tumours

## Abstract

**Background**: Chronic kidney disease (CKD) poses a significant global health challenge, affecting approximately 10% of the population. Patients with an acquired solitary kidney (ASK) from nephrectomy face elevated risks for CKD progression because of the increased functional demand on the remaining kidney. This study aims to identify risk factors for CKD progression in patients with a surgical ASK, highlighting the challenges faced by this population. **Methods**: This study retrospectively examined factors associated with renal function decline in 115 ASK patients who underwent nephrectomy for various pathologies, including renal tumours, urothelial tumours, and trauma. Follow-up assessments were conducted at 1, 12, 24, and 36 months post-nephrectomy, examining glomerular filtration rate (eGFR) and other renal function markers. Preoperative and postoperative data were analysed, with creatinine and eGFR measurements taken preoperatively, immediately postoperatively, and at all follow-up intervals. **Results**: The results of this study, which revealed that hypertension, diabetes mellitus, and preoperative kidney stones in the remaining kidney were significantly associated with accelerated CKD progression, with odds ratios of 2.7, 3.5, and 3.2, respectively, underscore the need for further research in this area. Although dyslipidaemia was observed in most patients (60%), its association with CKD risk did not reach statistical significance (*p* = 0.06). **Conclusions**: Our study highlights the critical need for ongoing urological assessment and tailored management strategies for patients with a solitary kidney following nephrectomy. By identifying key risk factors associated with renal function decline, we emphasise the importance of proactive monitoring and intervention to enhance long-term outcomes in this vulnerable population.

## 1. Introduction

Chronic kidney disease (CKD) significantly impacts global health, affecting over 800 million people worldwide, or approximately 10% of the global population, and has severe implications for patient survival and quality of life. The primary factors associated with the onset and progression of CKD are well established, with metabolic disorders (such as diabetes) and cardiovascular diseases (like hypertension) being frequently implicated [1,2].

A particular area of focus in recent years has been on patients with an acquired solitary kidney (ASK), especially those who have undergone nephrectomy for renal transplantation. Despite the morphologically and functionally normal state of the remaining kidney in most kidney transplant donors, this population exhibits a significantly higher risk of developing CKD compared with individuals who have not undergone nephrectomy for organ donation [3]. While unilateral renal agenesis is a congenital condition, the most common causes of ASK are surgical interventions such as nephrectomy for renal carcinoma, urothelial tumours, or severe trauma [4].

Although extensive data are available on risks and follow-up for solitary kidney patients post-transplantation, less attention is given to those with ASK from nephrectomy due to other pathologies, like tumours or trauma.

Based on Global Cancer Observatory data from 2022, approximately 145,800 new cases of kidney neoplasms are diagnosed in Europe each year, representing 2% of all cancer diagnoses, while kidney trauma constitutes approximately 10% of all hospitalisations for abdominal injuries [5,6]. These patients, who may have specific and potentially overlooked follow-up needs, often receive less focused attention, potentially leaving certain risk factors and preventive strategies underexplored. As a result, there is a need for increased awareness and targeted research to optimise long-term care for surgical solitary kidney patients outside the transplantation setting. Given the lack of data in the literature, our study aims to identify factors associated with renal function decline and highlight the need for targeted, long-term monitoring and management strategies to prevent renal complications in these patients.

## 2. Materials and Methods

This retrospective study included patients diagnosed and treated at the Urology and Renal Transplant Clinic, Dr. C.I. Parhon Hospital in Iasi, Romania, between January 2019 and January 2021. The study specifically focused on patients with ASK resulting from nephrectomy performed for renal carcinoma, upper urothelial tract carcinoma, severe renal trauma, lithiasic pyonephrosis, or non-functional kidneys, as confirmed by scintigraphy.

Inclusion criteria were age over 18 years and diagnoses such as renal cancer, upper urothelial tract carcinoma, severe renal trauma, lithiasic pyonephrosis, or non-functional kidneys documented by scintigraphy.

For exclusion criteria, patients with a prior diagnosis of CKD were excluded to isolate the impact of a solitary kidney on CKD progression. Based on the inclusion and exclusion criteria, patients were consecutively recruited from the Urology and Renal Transplant Clinic database at Dr. C.I. Parhon Hospital. Additionally, those who died within the three-year follow-up period or were lost to follow-up were excluded to ensure consistency in long-term data collection and outcome assessment.

Laboratory tests, including blood and urine analyses, were conducted for all patients. Serum and urine samples were processed using automated biochemistry analysers (Architect C4000 (Abbott, Chicago, IL, USA) and Urisys 1800 (Roche, Basel, Switzerland)). The following biohumoral values were analysed: haemoglobin, ionogram, creatinine, urea, cholesterol, triglycerides, blood sugar, and inflammatory parameters.

Body mass index (BMI) was calculated as weight (kg) divided by height squared (m²). 

Hypertension was defined as a systolic blood pressure ≥ 140 mmHg and/or diastolic blood pressure ≥ 90 mmHg or current use of antihypertensive medication. Dyslipidaemia was determined based on the presence of total cholesterol ≥ 200 mg/dL, LDL cholesterol ≥ 130 mg/dL, HDL cholesterol < 40 mg/dL (for men) or <50 mg/dL (for women), triglycerides ≥ 150 mg/dL, or current use of lipid-lowering medication.

The imaging evaluation, including ultrasonography and computed tomography, was conducted preoperatively to assess the condition of the remaining kidney and the primary pathology necessitating nephrectomy. Preoperative and postoperative laboratory data were analysed, with additional assessments conducted at 1 month and subsequently at 12, 24, and 36 months.

The eGFR was estimated using the CKD-EPI (Chronic Kidney Disease Epidemiology Collaboration) equation, which integrates serum creatinine levels, age, sex, and race, expressed in mL/min/1.73 m². A rapid decline was defined as a decrease in eGFR ≥ 30% from baseline.

All data regarding the patients’ anthropometric parameters, laboratory analyses, and secondary diagnoses were retrieved from the hospital’s information system. Upon discharge, patients were provided with informational materials in the form of brochures concerning lifestyle management and recommendations for nephrological follow-up.

The informed consent process adhered to the principles of the Declaration of Helsinki and received formal approval from the Ethics Committee of Dr C.I. Parhon Clinical Hospital (approval number: 9491/21 October 2024).

Statistical analyses were performed using SPSS software version 27 (IBM Corp., New York, NY, USA). Continuous variables were reported as means ± standard deviations, while categorical variables were presented as frequencies and percentages. Group comparisons were conducted using the χ² test for categorical variables, with Fisher’s exact test applied in small sample sizes (fewer than five cases per group). A *p*-value < 0.05 was considered statistically significant. Logistic regression analysis was performed to identify factors associated with a decline in eGFR, defined as a decrease of ≥30% from baseline. The dependent variable in the model was a rapid decline in eGFR (yes/no). The independent variables included gender, age, preoperative creatinine, the presence of comorbidities (hypertension, dyslipidaemia, renal lithiasis on the remaining kidney, cysts in the remaining kidney) and the patient’s habits (active smoking or alcohol consumption). The model was constructed using a stepwise forward selection method, with variables included if *p* < 0.05. Results were reported as odds ratios (OR) with 95% confidence intervals (CIs). Statistical significance was set at *p* < 0.05.

## 3. Results

After applying the inclusion and exclusion criteria, 115 patients aged 19 and 82 (average age 59.08 ± 17 years) who required nephrectomy were included in this study. Table 1 summarises the demographic characteristics, primary associated pathologies, and condition of the remaining kidney among patients in this study. The cohort was predominantly male (60%), with high rates of hypertension (44.3%), diabetes (53.9%), and dyslipidaemia (60%). Additionally, a significant proportion reported smoking (53%) and alcohol consumption (47%), factors with potential implications for renal outcomes. Preoperative evaluation of the remaining kidney revealed that 75.7% of patients had a normal kidney, 15.6% presented non-obstructive lithiasis, and 8.7% had cystic formations. The primary indications for nephrectomy were renal tumours (48.7%) and pyonephrosis (22.6%), followed by urothelial tumours (15.7%) and, to a lesser extent, trauma (5.2%) and congenital hydronephrosis (1.7%).

Table 2 presents a comparative analysis of creatinine levels measured at multiple checkpoints—preoperatively, immediately postoperatively, and at follow-up intervals of 1, 12, 24, and 36 months. The analysis was conducted for the entire patient cohort and each pathology subgroup. Across the sample, creatinine levels showed an initial postoperative increase (mean 1.47 mg/dL) compared to preoperative values (mean 1.27 mg/dL), with a mean of 1.71 mg/dL recorded at 36 months, reflecting statistically significant variability among subgroups. A comparative analysis of creatinine progression over time for the entire group also demonstrated a substantial decline, particularly at the 12, 24, and 36-month checkpoints.

Patients with emphysematous pyelonephritis had notably high creatinine levels preoperatively (mean 2.92 mg/dL) and postoperatively (mean 3.32 mg/dL).

Table 3 shows the statistical comparisons related to the creatinine levels reported for the entire group of patients and each pathology. Thus, a significant increase in creatinine levels was found in the case of neoplastic pathologies, while for non-neoplastic pathologies, except emphysematous pyelonephritis, no statistically significant creatinine increase was observed at 12, 24, and 36 months (*p* ≥ 0.05).

In Table 4, we analysed the evolution of eGFR values. The overall mean eGFR was 70.67 mL/min/1.73 m², with the TR group showing the highest mean (77.32), while at the opposite pole, the patients diagnosed with EP had the lowest values. Postoperative eGFR generally decreased, yet the CH group maintained higher postoperative eGFR. At one month, eGFR values remained below preoperative levels in most groups, pointing to a period of renal adaptation. Over 12 to 36 months, eGFR showed a declining trend, especially for neoplastic pathologies.

Table 5 provides a comprehensive statistical comparison of eGFR across the entire patient cohort, supplemented by detailed subgroup analyses for each pathology. The findings reveal significant changes in groups such as RT and UT, underscoring the differential trajectories of renal function decline over time and emphasising the varied impact of underlying pathologies on long-term renal outcomes.

Table 6 analyses the risk factors through a logistic regression analysis. Logistic regression analysis was performed for the entire cohort of patients with ASK to identify predictors of CKD progression. The results indicate that hypertension (OR = 2.7) and diabetes (OR = 3.5) are significantly associated with the onset and progression of CKD. Additionally, preoperative lithiasis in the remaining kidney strongly correlates with CKD (OR = 3.2). In contrast, factors such as gender, alcohol consumption, smoking, and preoperative cysts in the remaining kidney do not appear to significantly influence CKD outcomes, suggesting they may be less relevant predictors in this context. Furthermore, logistic regression analysis revealed that age is a significant predictor (*p* = 0.014), with the odds of CKD increasing by 3.4% for each additional year of age. However, preoperative creatinine did not significantly affect the outcome (*p* = 0.416).

## 4. Discussion

Our study highlights information regarding renal function in patients with ASK following nephrectomy for various pathologies. While extensive data exist in the literature regarding outcomes in transplant-related solitary kidney patients, fewer studies have focused on the risks and prognostic factors for those undergoing nephrectomy due to renal tumours, urothelial tumours, or trauma [4]. Our analysis revealed that patients with ASK, despite having a seemingly normal remaining kidney, show a progressive decline in renal function over time. This highlights the unique vulnerabilities of losing one kidney and the increased renal workload on the remaining kidney. These findings are consistent with prior studies suggesting that ASK patients are at elevated risk for CKD, even when the remaining kidney appears healthy initially.

CKD is a clinical condition defined by the presence of structural or functional abnormalities in the kidneys, evidenced by markers of kidney damage or a sustained reduction in eGFR for at least three months, irrespective of aetiology [7]. Kidney surgery is a significant risk factor for the development of acute kidney injury (AKI) and CKD. Post-surgical nephron loss triggers compensatory hyperfiltration, which can activate maladaptive mechanisms, ultimately accelerating further nephron loss over time. The occurrence of even mild to severe AKI in the perioperative period is a well-established predictor of poor kidney outcomes, ranging from varying degrees of CKD to end-stage kidney disease (ESKD). Therefore, identifying reliable markers to effectively stratify patients and accurately identify those at higher risk is crucial [8,9].

In the context of these diverse CKD trajectories, patients with ASK due to nephrectomy face unique challenges. The increased functional burden on the remaining kidney, combined with risk factors such as hypertension and metabolic disturbances, makes this subgroup particularly vulnerable to progressive renal decline.

While providing essential intervention for various conditions—such as oncological control, trauma, or pyonephrosis—surgical reduction of functional kidney parenchyma can lead to diminished kidney function, often reflected in a declining estimated eGFR. Consequently, many patients with previously normal kidney function before nephrectomy may meet the eGFR criteria for chronic kidney disease postoperatively. In our study, most patients (64.4%) presented with neoplastic conditions, including 48.7% with renal parenchymal and 15.7% with urothelial tumours. Across these groups, average creatinine levels were comparable, except in patients diagnosed with emphysematous pyelonephritis at admission, who exhibited elevated creatinine levels due to the severe septic state associated with this condition. We found that, during the surveillance period, the creatinine levels of the patients included in this study were variable. Still, these were more evident, especially in the case of oncological pathologies, which is also true for the eGFR.

Our findings align with the study by Ellis et al., which demonstrated that patients with ASK are at increased risk of CKD progression and eventual kidney failure due to the heightened functional demands on the remaining kidney. Their study reported that, among the cohort with a single kidney, 10% progressed to kidney failure or required kidney replacement therapy, while 17% experienced mortality over the follow-up period [9].

Regarding the renal function of patients with renal tumours, prior research from tertiary care centres has shown that partial nephrectomy (PN) is associated with a lower incidence of postoperative serum creatinine elevation and a reduced risk of developing new-onset CKD compared to radical nephrectomy (RN). This evidence underscores the potential protective effects of preserving renal parenchyma through PN, which may help maintain better kidney function. Furthermore, findings from population-based studies support these observations, indicating that PN-treated patients generally experience more favourable renal outcomes, including slower rates of renal function decline and fewer kidney-related complications than those treated with RN [10,11]. Our results regarding the onset and progression of chronic kidney disease in patients with nephrectomies for kidney tumours are also supported by other studies in the literature. Huang et al. also compared the dynamics of renal function in patients with renal tumours operated by PN or RN. They concluded that patients who undergo RN are more exposed to the progression of chronic kidney disease, especially those with an eGFR impairment before surgery [12].

A study conducted by Velmahos et al. evaluated the potential risk of renal failure following nephrectomy for trauma, suggesting a significant association between nephrectomy and subsequent renal impairment. Their findings indicate that trauma patients who undergo nephrectomy may face an elevated long-term risk of renal failure, likely due to the abrupt loss of nephron mass and the increased functional burden placed on the remaining kidney [13]. In contrast, in our study, we did not observe significant changes in creatinine levels or eGFR among patients who underwent nephrectomy following trauma. This discrepancy may be attributed to the demographic characteristics of our study cohort, which consisted primarily of young, healthy individuals without comorbidities. The relative youth and absence of underlying health issues likely afforded a greater adaptive capacity for renal function post-nephrectomy, allowing the remaining kidney to compensate more effectively than in older or comorbid populations.

Our logistic regression highlighted several elements that can significantly contribute to the probability of CKD in patients with ASK. For example, the presence of arterial hypertension in the patients in our research was associated with a 2.7 times higher likelihood of eGFR degradation, a statistically significant result.

In individuals with ASK, pathologies such as diabetes mellitus, hypertension, and dyslipidaemia play a critical role in the progression of CKD and end-stage renal disease (ESRD). These metabolic conditions are similar to those affecting the general population but can have an accelerated impact on individuals with a reduced renal reserve. Hypertension, for instance, places increased pressure on the remaining kidney, potentially leading to glomerular hyperfiltration and accelerated decline in renal function. Studies have shown that when blood pressure is not optimally controlled, patients with a solitary kidney face a higher risk of CKD progression, underscoring the importance of stringent blood pressure management in this group [14,15,16]. In a retrospective analysis evaluating the evolution of renal function in patients with a single kidney, Wang et al. stated that the prevalence of hypertension and renal insufficiency was 36.9% and 35.4%, respectively, and that approximately one-third of patients with a single kidney have hypertension [17,18]. Arterial hypertension is widely documented as a critical factor in the onset and progression of chronic kidney disease CKD, primarily due to the elevated intraglomerular pressure it generates. Studies highlight that this persistent pressure induces glomerular hypertension and hyperfiltration, ultimately leading to structural damage manifesting as proteinuria and progressive renal function decline. Additionally, research points to activating the renin–angiotensin–aldosterone system (RAAS) as a contributing pathway, where RAAS-driven vasoconstriction, sodium retention, and fluid overload exacerbate renal stress, further accelerating CKD progression [19,20].

In our research, diabetes mellitus is the factor most strongly associated with the likelihood of developing CKD, with the risk being 3.5 times higher in diabetic patients compared to non-diabetic patients. Furthermore, diabetes mellitus is one of the well-established factors implicated in the progression of chronic kidney disease, and numerous studies in the literature support our data. Diabetes further complicates outcomes in solitary kidney patients, as chronic hyperglycaemia damages renal microvasculature, accelerating eGFR decline and potentially leading to diabetic nephropathy [14]. The risk of ESRD increases significantly when diabetes is present, primarily if it is poorly controlled. In most Western countries, diabetic nephropathy stands as the primary cause of CKD [17,21]. In addition to the diabetic nephropathy that leads to a decline in renal function, diabetic patients also experience glomerular hyperfiltration. Several theories have been proposed to explain renal hyperfiltration in the context of diabetes mellitus [22].

Additionally, dyslipidaemia contributes to vascular inflammation and atherosclerosis, affecting the kidney’s filtration capacity over time [23]. In some studies, the presence of dyslipidaemia is an independent factor in the occurrence and progression of chronic kidney disease. For example, Alvarez et al. stated that patients with pathological levels of triglycerides have a fivefold higher risk of CKD. In contrast, patients with pathological levels of cholesterol have a sixfold higher risk [24]. In our research, a significant proportion of nephrectomised patients were diagnosed with dyslipidaemia (60%). However, our data did not show statistically significant evidence for dyslipidaemia’s role in CKD development. However, the association was close to significance (*p* = 0.06), suggesting a risk of approximately five times higher than in patients without lipid metabolism disorders.

An important aspect considered in our study was the preoperative assessment of the condition of the remaining kidney. As observed, 25.2% of patients had a personal history of urinary stones. At the time of nephrectomy, 15.6% had non-obstructive stones, documented through imaging, in the remaining kidney, while 8.7% presented with uncomplicated renal cysts. Although ESRD directly attributed to kidney stones is relatively low, with an estimated prevalence of 3.2% among patients initiating maintenance haemodialysis, kidney stones may still play a contributing role in the development and progression of CKD [19]. Studies focused on this issue indicate that patients with kidney stones have roughly twice the risk of developing chronic kidney disease compared to the general population [20]. The mechanism by which kidney stones pose a risk factor for CKD is multifactorial. On the one hand, the chronic inflammation associated with the presence of stones leads to a loss of nephron mass. On the other hand, certain underlying conditions that predispose to stone formation—such as primary hyperoxaluria, cystinuria, and Dent disease—are known to cause chronic renal impairment [25,26]. A particular case can be made for patients with uric acid stones, who are more prone to the onset and progression of CKD compared to those with other stone types. This increased risk is attributed partly to the higher prevalence of diabetic nephropathy in these patients and partly to the high recurrence rate associated with this biochemical subtype of stones [27,28]. Our findings are consistent with data reported in the literature, suggesting that the presence of kidney stones in the remaining kidney significantly elevates the risk of CKD development. Specifically, patients with renal calculi on the solitary kidney demonstrated a 3.2-fold increased likelihood of progressing to CKD compared to non-stone-forming patients.

In patients with ASK, heightened vigilance and active monitoring are essential to identify risk factors that may contribute to the onset and progression of CKD, particularly given the increased risk of advancing to ESKD. Among the key non-pharmacological interventions to slow CKD progression, dietary protein reduction has shown promising benefits. Evidence supports that a high-protein diet correlates with an elevated risk of CKD and may accelerate disease progression because of its impact on intraglomerular dynamics [29,30]. The importance of involving a nutritionist in the management of patients with CKD, particularly those with a solitary kidney, cannot be overstated. Nutritionists are important in guiding dietary adjustments that align with each patient’s specific renal function and proteinuria levels, ensuring that nutritional interventions are safe and effective [29]. In our study, many patients were referred for ongoing supervision with nutritionists and nephrologists to create personalised dietary plans and support long-term adherence to recommended dietary changes. Additionally, we provided patients with informational brochures and educational materials that outlined critical nutritional principles, including the benefits of a low-protein diet, plant-based fibre sources, and the Dietary Approaches to Stop Hypertension diet. This multidisciplinary approach aimed to empower patients with knowledge and support, enabling them to participate in actively managing their condition and mitigating CKD progression.

This study focuses on the progression of CKD in patients with ASK due to nephrectomy for diverse pathologies, distinguishing it from prior research predominantly centred on the transplant or congenital solitary kidneys. By providing a detailed longitudinal follow-up of renal function over 36 months, the study identifies key risk factors such as hypertension, diabetes, and contralateral remaining kidney lithiasis, emphasising the differential impact of underlying pathologies like renal and urothelial tumours. These findings highlight the need for tailored, pathology-specific management strategies, offering new insights to improve long-term outcomes in this vulnerable population.

As a retrospective, single-centre study, the findings may need more generalisability because of local practices, patient demographics, and short follow-up durations (36 months), potentially limiting insights into the long-term progression of CKD in patients with ASK. While key metabolic factors like hypertension and diabetes were considered, detailed lifestyle and dietary data were unavailable. Another limitation of this study was the inability to measure proteinuria in all patients, which prevented this parameter from being included in the analysis.

## 5. Conclusions

Our study underscores the heightened risk of CKD progression in patients with ASK, particularly among patients with comorbid conditions, such as hypertension, diabetes mellitus, and urinary stones. These findings emphasise the critical importance of adopting an integrative, multidisciplinary approach to patient management, leveraging the combined expertise of nephrology and urology specialists. Such a collaborative framework facilitates the development of individualised, pathology-specific management strategies to preserve renal function. This comprehensive approach can significantly improve long-term outcomes and quality of life for this high-risk population by addressing the complex interplay of underlying comorbidities and enhancing early detection and intervention strategies.

Our study emphasises the increased risk of CKD progression in patients with an acquired solitary kidney, particularly those with coexisting conditions such as hypertension, diabetes, and dyslipidaemia. A key finding is the critical importance of vigilant monitoring of the contralateral kidney, especially in patients with a history of renal lithiasis, as they present an elevated risk of nephron loss and subsequent CKD. This highlights the essential role of a collaborative approach between nephrology and urology specialists, which can facilitate comprehensive management plans. Such multidisciplinary care, combined with proactive interventions, like tailored nutritional guidance and consistent nephrology follow-up, is vital to mitigating renal function decline and enhancing patient outcomes in this high-risk population.

## Figures and Tables

**Table 1:** Demographic characteristics of the patients, associated pathologies, lifestyle factors, and the condition of the remaining kidney.

Parameter		*n*	%
Gender	Male	69	60
	Female	46	40
Hypertension	Yes	51	44.3
	No	64	55.7
Diabetes	Yes	62	53.9
	No	53	46.1
Dyslipidaemia	Yes	69	60
	No	46	40
History of kidney stones	Yes	29	25.2
	No	86	74.8
Smoking	Yes	61	53
	No	54	47
Alcohol consumption	Yes	54	47
	No	61	53
Preoperative status of the remaining kidney	Normal	87	75.7
	Non-obstructive lithiasis	18	15.6
	Cysts	10	8.7
The primary indication for nephrectomy	Renal tumour	56	48.7
	Urothelial tumour	18	15.7
	Pyonephrosis	26	22.6
	Trauma	5	5.2
	Congenital hydronephrosis	2	1.7
	Emphysematous pyelonephritis	6	5.2
	Post percutaneous nephrolithotomy	1	0.9

**Table 2:** The evolution of creatinine values.

	Whole Sample (*n* = 115)	RT (*n* = 56)	UT (*n* = 18)	PYN (*n* = 26)	TR (*n* = 5)	CH (*n* = 2)	EP (*n* = 6)
Mean preoperative creatinine (mg/dL) (±SD)	1.27 (±0.45)	1.16 (±0.24)	1.27 (±0.22)	1.21 (±0.18)	1.04 (±0.21)	1.33 (±0.11)	2.92 (±0.51)
Mean postoperative creatinine (mg/dL) (±SD)	1.47 (±0.85)	1.42 (±0.80)	1.35 (±0.66)	1.35 (±0.63)	1.25 (±0.74)	1.05 (±0.16)	3.32 (±0.89)
Mean creatinine 1 month postoperatively (mg/dL) (±SD)	1.29 (±0.37)	1.16 (±0.24)	1.27 (±0.25)	1.28 (±0.33)	1.34 (±0.37)	1.56 (±0.53)	2.19 (±1.09)
Mean creatinine 12 months postoperatively (mg/dL) (±SD)	1.74 (±0.50)	1.82 (±0.69)	1.83 (±0.43)	1.42 (±0.52)	1.42 (±0.78)	1.33 (±0.58)	2.88 (±1.04)
Mean creatinine 24 months postoperatively (mg/dL) (±SD)	1.72 (±0.69)	1.78 (±0.70)	1.82 (±0.38)	1.42 (±0.51)	1.45 (±0.80)	1.38 (±0.54)	2.84 (±1.02)
Mean creatinine 36 months postoperatively (mg/dL) (±SD)	1.71 (±0.70)	1.76 (±0.67)	1.88 (±0.59)	1.39 (±0.52)	1.37 (±0.73)	1.40 (±0.57)	2.73 (±0.91)

RT—renal tumour, UT—urothelial tumour, PYN—pyonephrosis, TR—trauma, CH—congenital hydronephrosis, EP—emphysematous pyelonephritis, SD—standard deviation.

**Table 3:** Statistical comparison of creatinine levels.

	Whole Sample	RT	UT	PYN	TR	CH	EP
Preoperative vs. immediate postoperative (CI 95%)	0.007 (−0.337–−0.054)	0.02 (−0.478–−0.027)	0.57 (−0.405–−0.233)	0.30 (−0.404–0.132)	0.54 (−1.063–0.633)	0.08 (−0.169–0.719)	0.38 (−1.489–0.689)
Preoperative vs. 1 month postoperative (CI 95%)	0.45 (−0.070–0.157)	0.01 (−0.295–−0.029)	0.97 (−0.083–−0.083)	0.39 (0.083–−0.009)	0.17 (−0.789–0.186)	0.57 (−3.983–3.513)	0.002 (1.041–2.334)
Preoperative vs. 12 months postoperative (CI 95%)	<0.001 (−0.529–−0.282)	<0.001 (−0.416–−6.413)	0.003 (−0.660–−0.166)	0.04 (0.415–0.009)	0.217 (−1.085–0.315)	0.91 (−6.354–6.224)	0.862 (−1.401–1.225)
Preoperative vs. 24 months postoperative (CI 95%)	<0.001 (−0.500–−0.282)	<0.001 (−0.794–0.416)	0.001 (−0.627–0.188)	0.02 (−0.398–− 0.031)	0.19 (−1.135–0.305)	0.92 (−5.963–5.853)	0.91 (−1.338–1.234)
Preoperative vs. 36 months postoperative (CI 95%)	<0.001 (−0.534–−0.283)	<0.001 (−0395–−6.317)	0.003 (−0.909–−0.217)	0.06 (−0.379–0.010)	0.26 (−1.001–0.341)	0.90 (−6.237–6.087)	0.96 (1.225–−0.049)

RT—renal tumour, UT—urothelial tumour, PYN—pyonephrosis, TR—trauma, CH—congenital hydronephrosis, EP—emphysematous pyelonephritis, CI—confidence Interval.

**Table 4:** The evolution of eGFR (CKD-EPI).

	Whole Sample (*n* = 115)	RT (*n* = 56)	UT (*n* = 18)	PYN (*n* = 26)	TR (*n* = 5)	CH (*n* = 2)	EP (*n* = 6)
Mean preoperative eGFR (±SD) (mL/min/1.73 m^2^)	70.67 (±19.6)	73.95 (±17.38)	71.16 (±11.56)	71.67 (±17.80)	77.32 (±17.65)	54.75 (±27.29)	23.13 (±8.90)
Mean postoperative eGFR (±SD) (mL/min/1.73 m^2^)	70.65 (±19.58)	62.53 (±27.82)	58.08 (±24.14)	64.06 (±25.99)	75.81 (±32.68)	80.90 (±12.93)	45.80 (±48.79)
Mean eGFR (±SD) (mL/min/1.73 m^2^) 1 month postoperatively	60.55 (±20.06)	57.70 (±18.59)	62.56 (±22.05)	60.89 (±20.69)	68.08 (±21.84)	56.09 (±20.66)	65.90 (±12.91)
Mean eGFR (±SD) (mL/min/1.73 m^2^) 12 months postoperatively	52.29 (±27.02)	50.13 (±24.22)	61.93 (±21.09)	60.01 (±31.49)	69.95 (±38.14)	80.30 (±13.57)	18.45 (±6.71)
Mean eGFR (±SD) (mL/min/1.73 m^2^) 24 months postoperatively	52.36 (±26.42)	51.87 (±24.17)	43.62 (±17.57)	59.33 (±30.78)	70.68 (±34.42)	58.75 (±29.90)	18.77 (±6.99)
Mean eGFR (±SD) (mL/min/1.73 m^2^) 36 months postoperatively	51.73 (±26.55)	49.02 (±23.89)	44.77 (±20.50)	60.77 (±31.37)	73.21 (±32.18)	53.55 (±23.96)	19.01 (±7.17)

RT—renal tumour, UT—urothelial tumour, PYN—pyonephrosis, TR—trauma, CH—congenital hydronephrosis, EP—emphysematous pyelonephritis, SD—standard deviation.

**Table 5:** Statistical comparison of eGFR levels.

	Whole Sample	RT	UT	PYN	TR	CH	EP
Preoperative vs. immediate postoperative (CI 95%)	0.22 (−0.009–0.042)	0.01 (2.741–20.095)	0.03 (0.732–25.538)	0.22 (−4.954–20.163)	0.92 (−38.033–41.049)	0.23 (−155.186–102.876)	0.24 (−67.047–21.681)
Preoperative vs. 1 month postoperative (CI 95%)	0.01 (5.112 –15.137)	<0.001 (9.506–22.984)	0.11 (−1.920–19.122)	0.04 (0.328–21.231)	0.39 (−16.003–34.486)	0.82 (−60.932–58.252)	0.002 (−60.543–−25.029)
Preoperative vs. 12 months postoperative (CI 95%)	0.001 (13.189–23.577)	<0.001 (16.546–31.090)	<0.001 (14.890–33.761)	0.09 (−2.106–25.402)	0.45 (−18.340–35.090)	0.53 (−392.759–341.659)	0.21 (−5.46–18.166)
Preoperative vs. 24 months postoperative (CI 95%)	0.001 (13.212–23.401)	<0.001 (14.784–29.366)	<0.001 (17.964–37.117)	0.06 (−0.723–25.402)	0.53 (−18.785–32.069)	0.93 (−517.907–509.897)	0.23 (−5.946–18.014)
Preoperative vs. 36 months postoperative (CI 95%)	0.002 (13.720–24.153)	<0.001 (17.748–32.106)	<0.001 (14.595–38.197)	0.10 (−2.487–24.281)	0.64 (−24.310–27.560)	0.97 (−459.341–461.731)	0.25 (−6.390–18.002)

RT—renal tumour, UT—urothelial tumour, PYN—pyonephrosis, TR—trauma, CH—congenital hydronephrosis, EP—emphysematous pyelonephritis, CI—confidence Interval.

**Table 6:** Multivariate logistic regression analysis of factors associated with CKD.

Parameter	B	*p*	OR	95% CI	
Gender	0.344	0.493	1.4	0.527	3.776
Age	0.034	0.01	1.03	1.007	1.063
Preoperative creatinine	0.374	0.416	1.45	0.590	3.580
Hypertension	0.995	0.03	2.7	1.066	6.862
Diabetes	1.255	0.01	3.5	1.350	9.114
Dyslipidaemia	0.850	0.06	2.3	0.951	5.758
Alcohol	0.323	0.48	1.31	0.561	3.397
Smoking	−0.202	0.66	0.81	0.332	2.012
Preoperative lithiasis on the remaining kidney	1.184	0.04	3.2	1.010	10.563
Preoperative cyst on the remaining kidney	−1.231	0.15	0.29	0.053	1.617

B—Beta coefficient, OR—Odds Ratio, 95% CI—95% Confidence Interval.

## Data Availability

The data supporting this study’s findings are available from the corresponding author upon reasonable request.
